# ^64^Cu-PSMA-617: A novel PSMA-targeted radio-tracer for PET imaging in gastric adenocarcinoma xenografted mice model

**DOI:** 10.18632/oncotarget.18276

**Published:** 2017-05-26

**Authors:** Xue-Di Han, Chen Liu, Fei Liu, Qing-Hua Xie, Te-Li Liu, Xiao-Yi Guo, Xiao-Xia Xu, Xing Yang, Hua Zhu, Zhi Yang

**Affiliations:** ^1^ Key Laboratory of Carcinogenesis and Translational Research (Ministry of Education), Department of Nuclear Medicine, Peking University Cancer Hospital and Institute, Beijing 100142, China; ^2^ College of Chemistry, Sichuan University, Chengdu 610064, China; ^3^ Russell H. Morgan, Department of Radiology and Radiological Science, Johns Hopkins Medical Institutions, Baltimore, Maryland 21287, United States

**Keywords:** prostate specific membrane antigen, neo-vasculature, gastric cancer, PET, 64Cu

## Abstract

Here, we report that it’s feasible for imaging gastric adenocarcinoma mice model with prostate-specific membrane antigen (PSMA) targeting imaging agents, which could potentially provide an alternate and readily translational tool for managing gastric adenocarcinoma. DKFZ-PSMA-617, a PSMA targeting ligand reported recently, was chosen to be radio-labeled with nuclide ^64^Cu. ^64^Cu-PSMA-617 was radio-synthesized in high radio-chemical yield and specific activity up to 19.3 GBq/µmol. It showed good stability *in vitro*. The specificity of ^64^Cu-PSMA-617 was confirmed by cell uptake experiments in PSMA (+) LNCaP cell and PSMA (-) PC-3 and gastric adenocarcinoma BGC-823 cells. Micro-PET imaging in BGC-823 and PC-3 xenografts nude mice was evaluated (*n =* 4). And the tumors were visualized and better tumor-to-background achieved till 24 h. Co-administration of N- [[[(1S)-1-Carboxy-3-methylbutyl]amino]-carbonyl]-L-glutamic acid (ZJ-43) can substantially block the uptake in those tumors. Dissected tumor tissues were analyzed by auto-radiography and immunohistochemistry, and these results confirmed the PSMA expression in neo-vasculature which explained the target molecular imaging of ^64^Cu-PSMA-617. All those results suggested ^64^Cu-PSMA-617 may serve as a novel radio-tracer for tumor imaging more than prostate cancer.

## INTRODUCTION

Globally, gastric cancer is the third most common cause of cancer-related death making up 9% of all the cases [1]. High incident rate of gastric cancer occurs in Asian countries, including Japan, South Korea and China, which account for around 60% of the cases worldwide [2–4]. Complete resection can achieve permanent control when the disease is at early stage, but due to its limited clinical symptom, over 80% of patients are initially diagnosed with advanced cancer with poor prognosis [5, 6]. Limitations exist in clinical available methods, such as endoscopic ultrasound (EUS), computed tomography (CT) and magnetic resonance imaging (MRI) [7], for accurately staging the disease, detecting metastatic lesions and monitoring recurrence after radical treatment. ^18^F-Fluorodeoxyglucose (^18^F-FDG), which has been generally applied for tumor detection with positron emission tomography (PET) [8], was reported with limited sensitivity and specificity for gastric cancer detection [9, 10]. Recently, novel molecular imaging methods have been evaluated under preclinical settings [11–14]. Here, we report that it’s feasible to image gastric adenocarcinoma with prostate-specific membrane antigen (PSMA) targeting imaging agents, which could potentially provide an alternate and readily translational tool for managing gastric cancer.

PSMA is one of the most intensively investigated receptor for targeting and imaging metastatic prostate cancer. It is a zinc-dependent metallopeptidase located on the membrane surface catalyzing the hydrolysis of N-Acetylaspartylglutamic acid (NAAG) and its analogues [15–18]. With its limited normal tissue expression [19], PSMA is an ideal marker for metastatic diseases imaging. Several agents have reached clinic for prostate cancer staging [20–25]. Besides prostate cancer, PSMA over-expression has been reported in a variety of solid tumor neo-vasculature [26–28]. As reported, 66% of gastric carcinomas were detected with PSMA expression in tumor-associated neo-vasculature by analyzing the tissue specimens from the primary site of 119 patients [29]. It potentially provides another aspect for staging gastric cancer with existing PSMA targeting agents in clinic.

DKFZ-PSMA-617, a recently reported PSMA targeting ligand, was chosen for our pilot study [30, 31]. With the 1,4,7,10-tetraazacyclododecane-1,4,7,10-tetraacetic acid (DOTA) metal chelator, this ligand has been applied for labeling with several radioisotopes, including ^68^Ga [32], ^64^Cu [33] and ^177^Lu [34, 35], for imaging and treating prostate cancer in clinical studies. They demonstrated favorable *in vivo* pharmacokinetic property. We applied ^64^Cu-PSMA-617 for this initial investigation. [36]. The relatively long-lived isotope ^64^Cu (12.7 h) enabled us to carry out the imaging and auto-radiography studies conveniently. We demonstrate it is feasible to detect gastric adenocarcinoma in the xenograft BGC-823 tumor model by ^64^Cu-PSMA-617 PET imaging. Auto-radiography and immunohistochemical stain studies showed the uptake of the radio-tracer is from PSMA expressed in tumor-associated neo-vasculature. With the similarity among these DKFZ-PSMA-617 based agents, it could be easily adapted to imaging gastric adenocarcinoma.

## RESULTS

### Radio-synthesis and quality control

**Figure 1:**
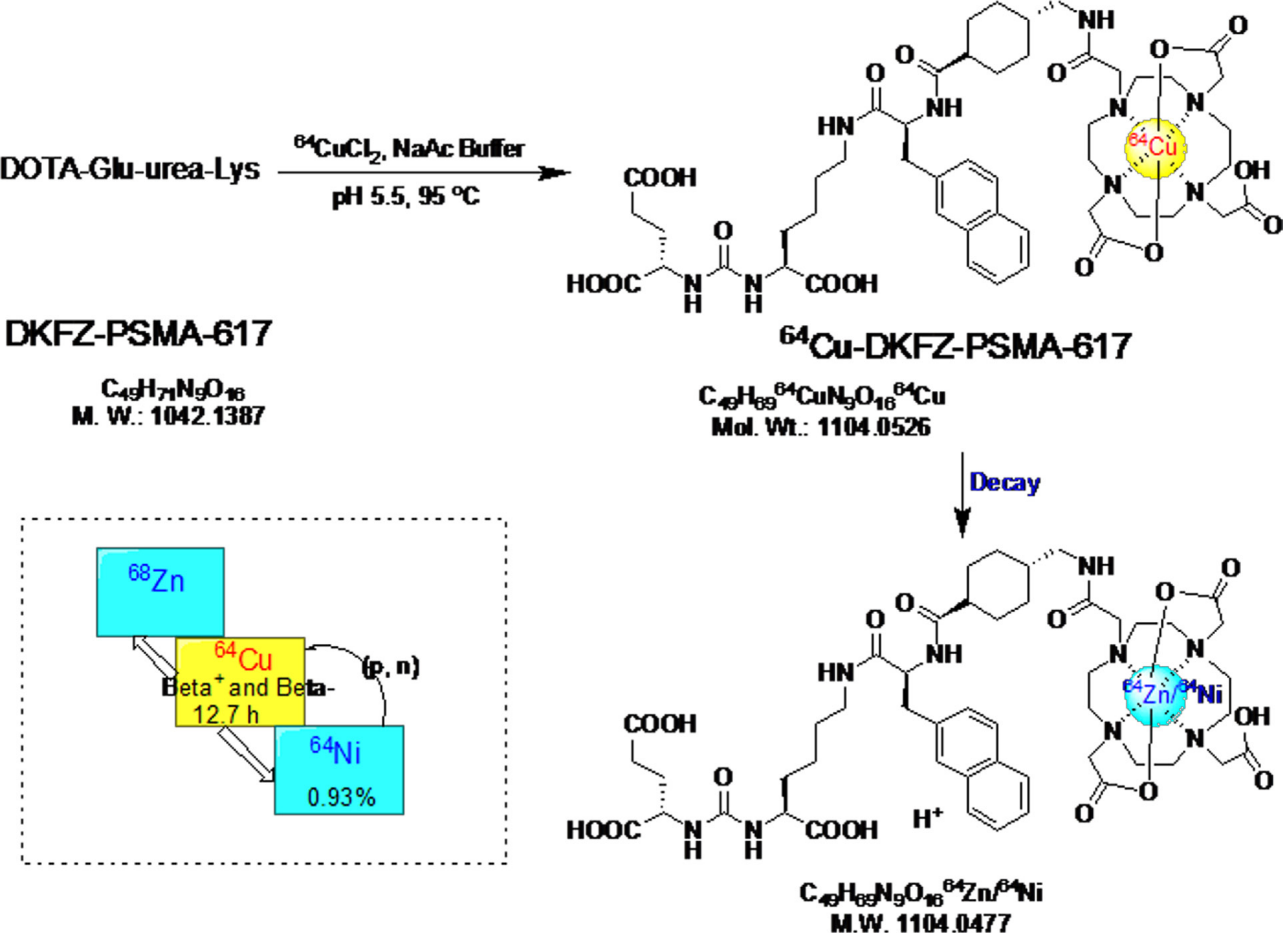
The radio-labeling and decay of ^64^Cu-PSMA-617 The dotted line box on the lower left gives the decay properties of related nuclide.

As it is depicted in Figure 1, ^64^Cu-PSMA-617 was synthesized by incubating ^64^CuCl_2_ with the ligand in a pH = 5.5 buffer at 95°C for 10 min. After a Sep-Pak C18 cartridge purification, the radio-tracer was obtained in over 99% radio-chemical purification yield. There was no free ^64^Cu in the product, as demonstrated by radio-High Performance Liquid Chromatography (HPLC) (Supplementary Figure 1). The specific activity of ^64^Cu-PSMA-617 was 2.96–6.74 GBq/µmol (*n =* 4). The electric charge of ^64^Cu-PSMA-617 was tested to be neutrality (Supplementary Figure 2).

The quality control of ^64^Cu-PSMA-617 was tested and shown in Table 1. ^64^Cu-PSMA-617 had the purity of more than 98% tested by radio-TLC and over 99% by radio-HPLC. There was no ethanol and few endotoxins in the ^64^Cu-PSMA-617 product. The radio-tracer meets the quality requirement for preclinical study.

**Table 1:** Quality control (QC) of ^64^Cu-PSMA-617 for preclinical application

Parameter	QC specification	QC result
Appearance	Clear, colorless	Pass
Volume	0.5–1.0 mL	0.5 mL
Injection dose	18.5–37 MBq	18.537 MBq
pH	4.0–8.0	4.2
Radio-TLC	> 95%	> 98%
Radio-HPLC	> 95%	> 99%
Ethanol	< 5%	< 1%
Endotoxins	< 15 EU/mL	Pass
Sterility	Sterile	Pass
Specific activity	Not defined	2.96–6.74 GBq/µmol

### *In vitro* stability test

**Figure 2:**
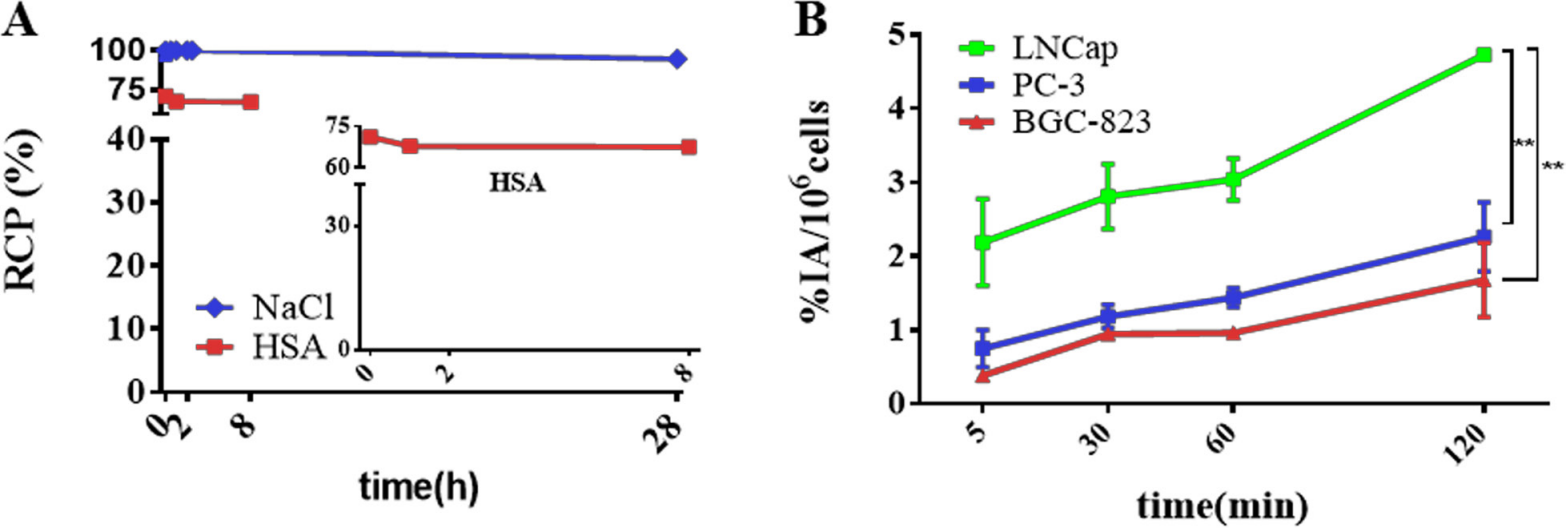
*In vitro* stability test and cell uptake analysis of ^64^Cu-PSMA-617 **(A)** *In vitro* stability test of ^64^Cu-PSMA-617 in 0.9% saline (blue) and 5% HSA (red). **(B)** Cell uptake analysis of ^64^Cu-PSMA-617 tracer in PSMA(+) LNCaP, PSMA(–) PC-3 and BGC-823 cell lines at 5 min, 30 min, 1 h and 2 h. (**P < 0.01) (*n =* 4).

*In vitro* stability tests in 5% human serum albumin (HSA) and saline at 37.0°C were determined by radio-HPLC using the general method described in the experimental section. As depicted in Figure 2A, 94% radio-tracer remained intact after more than 24 h incubation in saline. Meanwhile, ^64^Cu-PSMA-617 in 5% HSA slightly decreased from 71.0% at 5 min to 67.0% at 8 h. These results demonstrate ^64^Cu-PSMA-617 has reasonable stability to be applied for clinical study.

### *In vitro* cell binding assay

Cell uptake experiments were performed on three kinds of tumor cells including PSMA (+) LNCaP, PSMA (-) PC-3 [37, 38] , BGC-823 cells as shown in Figure 2B. The uptake values of ^64^Cu-PSMA-617 in LNCaP cells were 2.2 ± 0.6 %, 2.8 ± 0.4%, 3.0 ± 0.3%, 4.7 ± 0.0% at 5 min, 30 min, 60 min, 120 min, respectively. At the same time, the uptake values of ^64^Cu-PSMA-617 in PC-3 and BGC-823 cells were 1.7 ± 0.5%, 2.3 ± 0.5% at 120 min. There was significant difference between LNCaP and PC-3, LNCaP and BGC-823 (*P <* 0.01), but not in PC-3 and BGC-823 (*P >* 0.05), which are consistent with very low PSMA expression in PC-3 and BGC-823 cells.

When blocked, the uptake of LNCaP was 2.9 ± 0.2% at 120 min. The 38% uptake reduction of ^64^Cu-PSMA-617 by the block of a known PSMA inhibitor N- [ [ [(1S)-1-Carboxy-3-methylbutyl]amino]-carbonyl]-L-glutamic acid (ZJ-43) [39] indicates that ZJ-43 ligands occupy the PSMA binding site at cell surface, and the binding sites are saturable (*P* < 0.01). At 120 min after co-administration of excess ZJ-43, the uptake values of PC-3 and BGC-823 remained at 1.2 ± 0.4% and 2.9 ± 0.7%, respectively (Supplementary Figure 3). There was no significant difference between normal uptake and blocked group in PC-3 and BGC-823 (*P >* 0.05), which further indicated low PSMA expression in those cells.

### *In vivo* toxicity

The toxicity of the radio-tracer in normal BALB/c mice was also evaluated. The mean administered activity was 18.5 MBq to normal mice (*n =* 4). There were no adverse or significant changes in vital signs observed from 0 h to 120 h after injection.

### Micro-PET imaging

**Figure 3:**
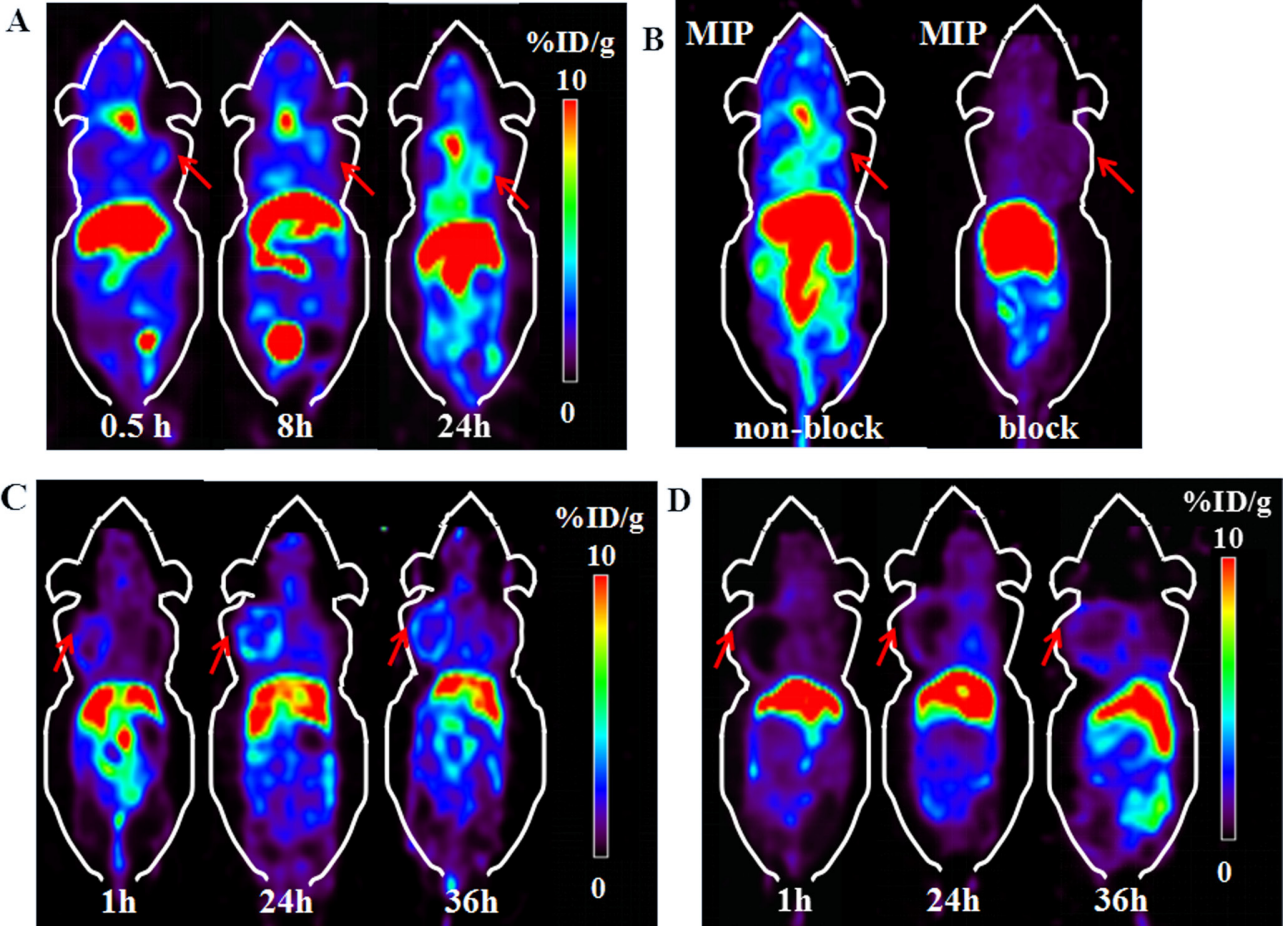
Micro-PET images of ^64^Cu-PSMA-617 in BGC-823 and PC-3 xenograft nude mice **(A)** Micro-PET images of ^64^Cu-PSMA-617 in BGC-823 gastric adenocarcinoma cell xenograft nude mice at 0.5 h, 8 h and 24 h after injection. **(B)** MIP images of ^64^Cu-PSMA-617 in BGC-823 bearing mouse with or without co-injection of blocker ZJ-43 (25 mg/kg) at 24 h. Red arrows indicate tumor. **(C)** Micro-PET images of ^64^Cu-PSMA-617 in PC-3 prostate cancer cell xenograft nude mice at 1 h, 24 h and 36 h after injection. **(D)** Micro-PET imaging of ^64^Cu-PSMA-617 co-injected with ZJ-43 (25 mg/kg) as blocker in PC-3 tumor. Red arrows indicate tumor.

PET images on nude mice bearing BGC-823 and PC-3 xenograft were performed on a micro-PET scanner. Representative decay-corrected images obtained at 0.5 h, 8 h and 24 h post injection in BGC-823 xenograft nude mice are shown in Figure 3A. The tumor organ could be clearly observed at 0.5 h after radio-tracer’s injection. The most significant uptake of the radio-tracer was in liver, and then salivary glands. The tumor uptake was moderate at 24 h after injection. Quantitative analyses of micro-PET images were conducted, region of interests (ROI) were drawn over main organs for each micro-PET scan. Mean pixel values were obtained as value of radio-activity concentration. For BGC-823 tumor xenograft model, the tumor/salivary ratios steadily increased over time from 0.34, 0.41 to 0.71 at 0.5 h, 8 h, 24 h respectively. The T/NT ratio also increased from 0.5 h to 24 h. The co-administration of excess PSMA inhibitor ZJ-43 (25 mg/kg) clearly decreased the BGC-823 tumor uptake (Figure 3B). This phenomenon were also monitored by micro-PET images of ^64^Cu-PSMA-617 in PC-3 xenograft nude mice (Figure 3C, 3D). PC-3 bearing mice also showed the similar distribution properties of ^64^Cu-PSMA-617. After 24 h post injection, good contrast images of tumor was obtained. The uptake of ^64^Cu-PSMA-617 in liver and kidney was relatively high, and the tumor showed moderate radio-tracer uptake at 24 h post injection. The radio-activity uptake was also observed in salivary glands. Uptake in both PC-3 tumor and salivary glands was substantially blocked by co-administration of excess ZJ-43. Quantification of PET images based on region of interest (ROI) was shown in Supplementary Figure 4.

### Biodistribution

Biodistribution studies were performed by tail vein injection of 0.74 MBq ^64^Cu-PSMA-617 radio-tracer in normal mice, BGC-823 and PC-3 xenografts mice. The retention of ^64^Cu-PSMA-617 in normal mice showed moderate blood clearance (Supplementary Figure 5), with 3.95 ± 0.75% ID/g at 1 h post injection, 3.62 ± 1.08% ID/g at 4 h, 1.91 ± 0.08% ID/g at 24 h post injection, respectively.

The log *P* value of ^64^Cu-PSMA-617 was determined to be −1.93 ± 0.13 from the octanol-water partition coefficient measurements, indicating high hydrophilic property of the radio-labeled peptide. Also, from the biodistribution study, the highest uptake of ^64^Cu-PSMA-617 was shown in kidney tissues, due to the metabolism through renal pathway as a hydrophilic compound and PSMA expression in kidney.

**Figure 4:**
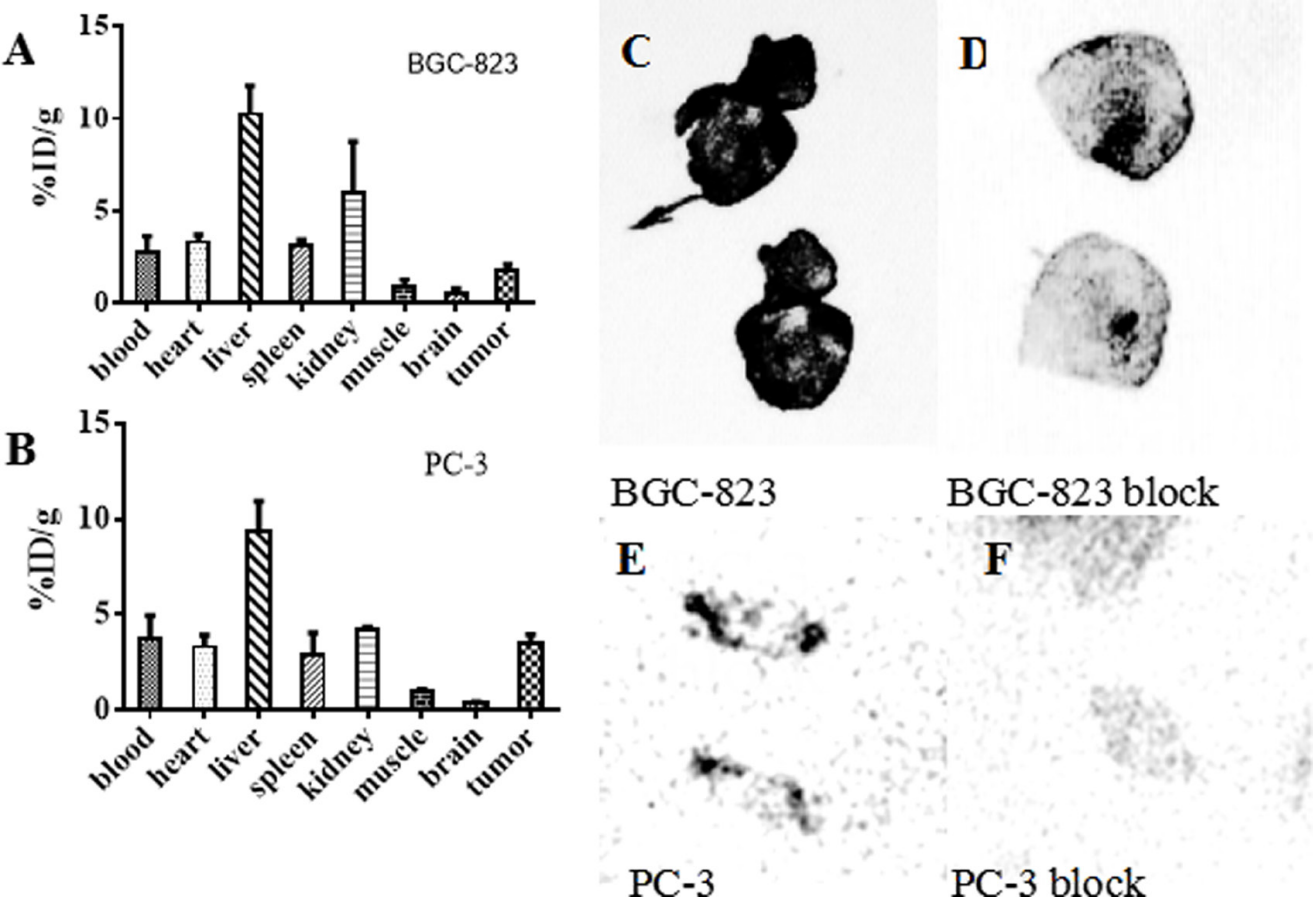
Biodistribution of ^64^Cu-PSMA-617 in tumor-bearing mice and the auto-radiography images of tumor tissues **(A, B)** Biodistribution of various organs of ^64^Cu-PSMA-617 after 24 h injection in BGC-823 and PC-3 bearing mice (*n =* 4). The error bar was calculated as the standard deviation. **(C–F)** Auto-radiography of BGC-823 and PC-3 tumor. C and D are the auto-radiography images of tumor sections (25 µm) obtained from BGC-823 tumor 24 h after injection of ^64^Cu-PSMA-617. E and F are tumor sections (25 µm) obtained from PC-3 tumor 72 h after injection of ^64^Cu-PSMA-617. The tumors periphery parts showed intense radio-activity (C, E) which decreased clearly in ZJ-43(25 mg/kg) co-injection group (D, F).

The biodistribution data showed that the liver had relatively high uptake, with 24.10 ± 2.34% ID/g at 4 h, and 8.13 ± 1.08% ID/g at 24 h. The uptake of ^64^Cu-PSMA-617 displayed good tumor accumulation, with 1.81 ± 0.29% ID/g and 3.47 ± 0.48% ID/g at 24 h in BGC-823 and PC-3 tumors, respectively. (Figure 4A, 4B)

### Auto-radiography and immunohistochemistry

Intense radio-activity was observed in the periphery position of BGC-823 and PC-3 tumor (Figure 4C, 4E). Also, the tumor uptake of ^64^Cu-PSMA-617 could be blocked by ZJ-43(25 mg/kg) (Figure 4D, 4F).

**Figure 5:**
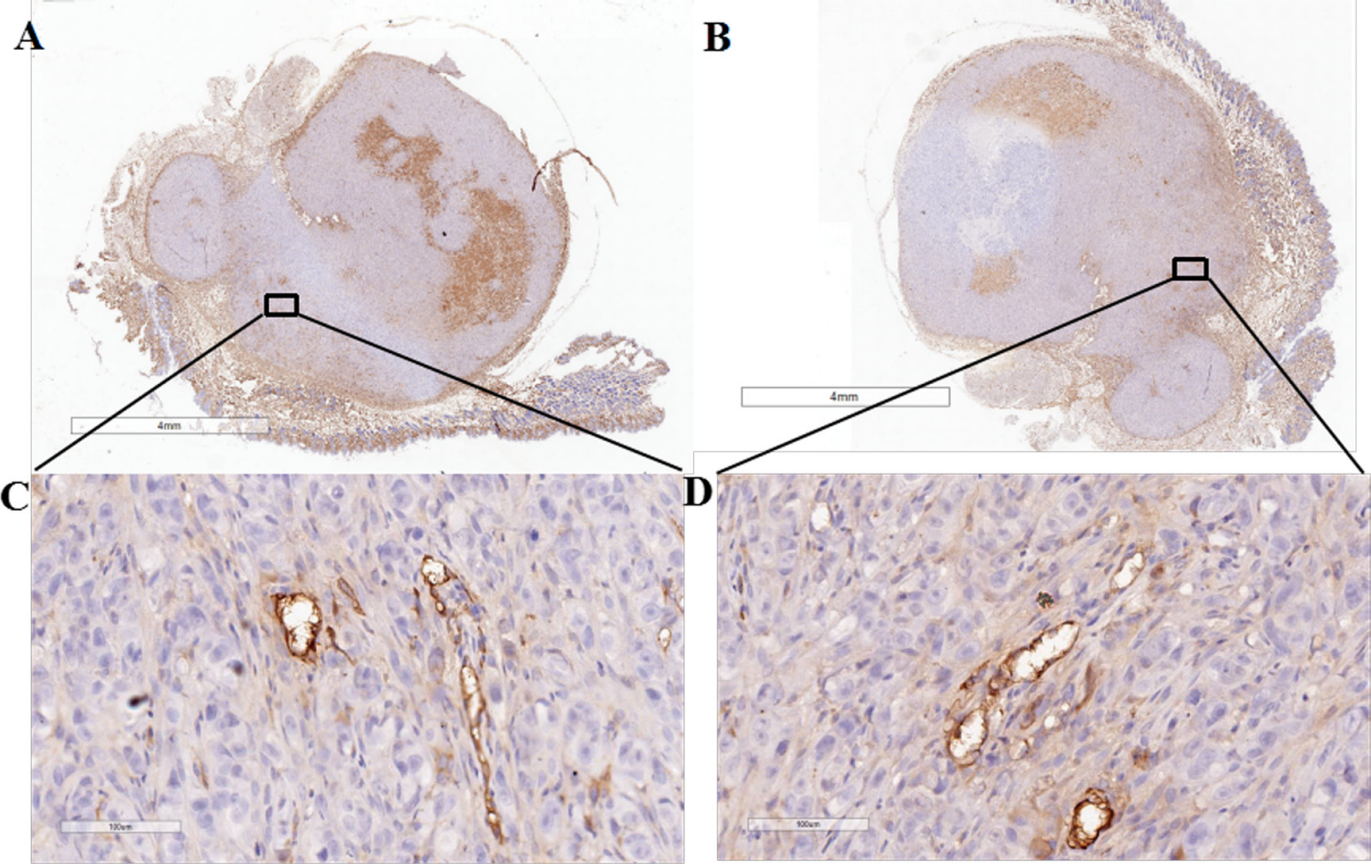
Immunohistochemical analysis of PC-3 tumor CD34 staining in **(A, C)** demonstrates staining of the tumor-associated vascular endothelium of tumor in the tumor periphery. Overlap of PSMA staining in the tumor vasculature is shown in **(B, D)**. The enlarged images of the squares in A, B (Scale = 4 mm) are shown in C, D (Scale = 100 μm).

To further confirm the PSMA expression in the neo-vasculature of PC-3 tumor, immunohistochemical experiment was performed to detect the PSMA expression in those dissect tumor tissues. CD34 receptor was chosen as a marker for vascular endothelial cells [40], and it has been proved to have high specificity to the parts of neo-vasculature, in comparison to the location of PSMA expression. Figure 5 showed overlap staining in the neo-vasculature of CD34 and PSMA, indicating the PSMA expression in neo-vasculature. In addition, PSMA staining showed higher intensity in the tumor periphery other than the center, consistent to auto-radiogram and micro-PET results.

## DISCUSSION

PSMA is a well-established target for prostate cancer imaging and radio-therapy. Several agents have been developed and studied clinically [41–44]. Among these agents, DKFZ-PSMA-617 was developed recently and showed good *in vivo* pharmacokinetic properties. With the metal chelator in the ligand, it’s suitable for ^68^Ga, ^64^Cu and ^177^Lu labeling for corresponding clinical diagnosis and therapy on PSMA over-expressing tumors [34, 35, 45]. ^64^Cu has a T_1/2_ of 12.7 h, with 17.8 % of decay through β^+^ and 38.4% of decay through β^-^, which potentially enables its theranostic applications. With the recent efforts, we are able to produce ^64^Cu in high quality with HM-20 cyclotron in China. With its relatively long half-life, we can carry out our experiment conveniently at later time points, when the radio-tracer clears from the non-specific organs and gives better specific targeting contrast. It may have the special value, when the receptor expression level is low, such as the neo-vasculature of gastric adenocarcinoma. The similarity between ^64^Cu and ^68^Ga/^177^Lu-PSMA-617, in principle, could allow switching for different purposes.

The ^64^Cu-PSMA-617 labeling and purification process took less than 20 min and resulted in over 99% radio-chemical yield. There was no free ^64^Cu in the product. The tracer had good specific activity of 2.96–6.74 GBq/µmol. Also, the radionuclide ^64^Cu labeled DKFZ-PSMA-617 product was further verified by matrix-assisted laser desorption ionization time of flight mass spectrometry (MALDI-TOF-MS) (Supplementary Figure 6). ^64^Cu-PSMA-617 met the quality standard for preclinical study and showed good biological safety in normal BALB/c mice. ^64^Cu-PSMA-617 showed great *in vitro* stability in both 5% HSA and saline at 37.0°C. It had the potential of application in clinical practice.

Cell uptake experiments were performed on PSMA (+) LNCaP, PSMA (-) PC-3 , BGC-823 cells. The uptake values of ^64^Cu-PSMA-617 in LNCaP cells increased with longer incubation time, and the uptake can be blocked by ZJ-43, indicating that ^64^Cu-PSMA-617 can bind to PSMA specifically. Meanwhile, the uptake values of ^64^Cu-PSMA-617 in PC-3 and BGC-823 cells were lower than LNCaP(*P <* 0.01) at 120 min and the uptake cannot be blocked by excess ZJ-43(*P >* 0.05), which verifies low PSMA expression in PC-3 and BGC-823 cells.

Micro-PET images were performed on nude mice bearing BGC-823 and PC-3 xenograft tumor. The tumors were clearly visible, and the radio-activity accumulated with time. Good contrast images of tumor were acquired at 24 h. Significant uptake were observed in liver and kidney, followed by the salivary glands and the tumor. Also, the co-injection of excess ZJ-43 clearly decreased the tumor uptake that indicated specific uptake of ^64^Cu-PSMA-617.

Biodistribution studies performed in normal mice, BGC-823 and PC-3 xenografts mice showed moderate blood clearance. ^64^Cu-PSMA-617 showed high uptake in kidney due to receptor expression, relatively high uptake in the liver because of the *in vivo* dislocation of ^64^Cu from the DOTA chelator of DKFZ-PSMA-617 [46]. It is found that PSMA is not only expressed in tumor-associated endothelium, but also in non-specific tissue especially in salivary glands [32]. Both BGC-823 and PC-3 bearing mice showed medium uptake of ^64^Cu-PSMA-617 in the salivary glands. There was low level of radio-activity in other non-specific organs such as muscle. Both BGC-823 and PC-3 tumors displayed good radio-tracer accumulation, and showed good tumor to muscle contrast. It demonstrated that the BGC-823 and PC-3 tumors had moderate uptake of PSMA-targeted radio-tracer, consistent with micro-PET study.

ZJ-43 can effectively block the PC-3 and BGC-823 tumors *in vivo*, but not PC-3 and BGC-823 cells *in vitro*. We supposed the difference in blocking results between *in vivo* and *in vitro* experiments reflected PSMA expression in tumor-associated neo-vasculature. The expression of PSMA in neo-vasculature of solid tumors has been reported in literatures [27, 47, 48]. For example, Godeiro’s group [49] have confirmed that PSMA is a unique biomarker specifically expressed by tumor-associated neo-vasculature but not expressed by normal vessels. Both BGC-823 and PC-3 xenograft tumor are typical solid tumors with abundant neo-vasculature, and may express PSMA. The co-administration of excess ZJ-43 would block the corresponding uptake of ^64^Cu-PSMA-617 in the *in vivo* studies.

To further confirm the PSMA expression in neo-vasculature of BGC-823 and PC-3 tumors, we carried the auto-radiography and immunohistochemical experiments to detect the PSMA expression in BGC-823 and PC-3 dissect tumor tissues. Low level of activity was observed in the tumor center, and intense radio-activity was observed in the periphery which can be blocked by ZJ-43 (25 mg/kg). From auto-radiogram, there was great uptake difference between non-block group and ZJ-43 block group. From auto-radiography study, high concentration of CD34 staining was found in the periphery of tumor, and lower level of staining appeared in the tumor center, consistent with auto-radiography images. The immunohistochemical study showed overlap staining of CD34 and PSMA staining in the neo-vasculature of tumor. We believe that the neo-vasculature in BGC-823 and PC-3 tumor periphery has certain degree of PSMA expression, which can be used as the target for molecular imaging.

In conclusion, we successfully radio-synthesized ^64^Cu-PSMA-617 with high purity and reproducibility. It showed specific binding affinity to PSMA over-expressing cells and tumors organs. Tumors can be observed as early as 1 h post injection to 24 h using micro-PET equipment. Our findings provide evidence that ^64^Cu-PSMA-617 has potential to image neo-vasculature in solid tumor, such as gastric cancer. It also serve as a radio-tracer for PET imaging for prostate cancer, which have been reported by Grubmüller B et al. [30].

Benefit from the long half life and β^-^ properties of ^64^Cu radio-nulide [50], ^64^Cu-PSMA-617 also has the potential to be used as a radio-therapy agent. However, compared with the good bio-distribution properties of several reported clinical or pre-clinical used PSMA-targeted tracers [22, 25, 42], DOTA chelator may not be a perfect selection for ^64^Cu chelation. Further improvements of ^64^Cu-PSMA-617 are needed.

## MATERIALS AND METHODS

### General

^64^Cu was produced *via* ^64^Ni (p,n)^64^Cu reaction at Beijing Cancer Hospital using the HM-20 cyclotron (specific activity of 5.6 GBq/µmol). RPMI 1640 medium, phosphate-buffered saline (PBS), fetal bovine serum (FBS), Penicillin, Streptomycin and 0.25%Typsin-EDTA(1X) were purchased from Invitrogen (CA, USA). Moxiz Z Cassette was purchased from ORFLO Technologies (Ketchum USA). ZJ-43 was purchased from Tocris Bioscience (Bristol, UK). Trifluoroacetic acid (TFA) and metal free concentrate HCl were purchased from Sigma-Aldrich (St. Louis, MO, USA). DKFZ-PSMA-617 was purchased from ABX company (Radeberg, Germany). Sep-Pak light C18 cartridge was purchased from Waters Company (Massachusetts, USA). MALDI-TOF was from Bruker (Bruker Daltonics, USA). The Agilent Technologies 1200 series of high performance liquid chromatography (HPLC) system was purchased from Agilent Technologies (California, USA) and applied for the characterization. HPLC condition was applied as follows. YMC-Pack ODS reversed-phase column (5 μm, 250 mm × 4.6 mm) was used and eluted with gradient 15/85/0.1 to 60/40/0.1 of H_2_O/Acetonitrile/TFA in 10 min. Instant thin-layer chromatography (ITLC) system used for analysis was AR-2000 radio-TLC Imaging Scanner (Washington DC, USA). ITLC-SG (silica gel) strips were purchased from Aligent Techbologies (Lake Forest, CA, USA) and cut to 1 cm in width. The ITLC was developed in 1/1 mixture of acetic acid ammonium and methanol.

### Cell lines and mice model

P53 mutant human gastric adenocarcinoma BGC-823 cell, LNCaP and PC-3 prostate cancer cells were obtained from China Infrastructure of Cell Line Resources and were maintained as shown in the guidelines. They were cultured in RPMI 1640 medium containing 10% (v/v) heat-inactivated FBS and 1%(v/v) Penicillin Streptomycin in a humidified incubator at 37°C with 5% CO_2_.

The BALB/c nude mice and normal BALB/c mice were purchased from Beijing Huafukang (HFK) Bioscience Co. Ltd. (Beijing, China). The BGC-823 tumor model was generated by subaxillary injection of 1 × 10^6^ tumor cells into the female nude mice. PC-3 tumor model was generated by subaxillary injection, of 3 × 10^6^ cells into male nude mice. All animal procedures were performed according to a protocol approved by the Peking University Cancer Hospital Animal Care and Use Committee. Tumor-bearing mice were used for micro-PET studies when tumors reached a size of approximately 0.5–1 cm^3^ in volume.

### Radio-synthesis

^64^Cu (3.7 MBq/µL diluted in 0.1 M HCl) was produced in HM-20 cyclotron (20 MeV, Sumitomo, Japan) *via* the ^64^Ni(p,n)^64^Cu nuclear reaction. 20 μL of a solution of the DKFZ-PSMA-617 (1.0 μg/μL) in a 1.5 mL Eppendorf cap was incubated with approximately 1.0 mL of ^64^Cu (180–280 MBq) in 0.1 M HCl at 90°C for 10 min. pH was checked to be 5.5. After the mixture was cooled to room temperature, the radio-tracer was analyzed by radio-HPLC. The crude reaction mixture was then transferred to a C18 sep-pak column. The C18 column was washed with 5.0 mL of deionized water. The final ^64^Cu-PSMA-617 product was eluted from the C18 column with 1.0 mL of 80% ethanol, and most of the ethanol was evaporated under a gentle stream of nitrogen. The remaining solution was diluted with 5.0 mL of 0.9% saline and sterile filtered. The radio-activity of the eluate was counted and calibrated against the standard solutions.

### Mass spectrometry analysis of decayed product

The decayed product of the radio-tracer ^64^Cu-PSMA-617 was further characterized by matrix-assisted laser desorption ionization time of flight mass spectrometry (MALDI-TOF-MS). After stored at 4°C for ten half lives of decay, the product was tested by MALDI-TOF-MS. The samples were diluted to 1.0 μg/ml by 0.1% TFA in water. Sinapinic acid was dissolved in acetonitrile/water/TFA (50/50/0.1) solution to a concentration of 10.0 mg/ml, and this solution was used for MALDI 1.0 μL solution, containing 1:1 mixture sample and matrix, was used for mass analysis. The spectrum was acquired in a positive linear mode and analyzed using the FlexAnalysis v3.0 software.

### *In vitro* stability study

The stability of ^64^Cu-PSMA-617 was evaluated in saline (pH = 7.4) at 0, 2 and 28 h post preparation. At least three sets of experiments were performed for each point. The HPLC condition used to analyze the stability was described in General experimental section.

### *In vitro* cell binding assay

24–48 h prior to the binding assay, confluent cells were detached and re-suspended, aliquots of 2 × 10^5^ cells were added to each well in the 24-well plate. Then ^64^Cu-PSMA-617(37 KBq ) was added to each well, the selected wells were co-administered with the inhibitor ZJ-43 for blocking to test the specific uptake of the radio-tracer. After 5, 30, 60 and 120 min incubation at 37°C, cells were washed twice with 1 mL ice-cold PBS, trypsinized with 0.3 nM NaOH buffer, the NaOH solutions were then collected for analysis. The values counted were all converted into percentage of incubated dose per million cells (%IA/10^6^cells). Experiments were repeated four times.

### Biodistribution

Normal BALB/c mice, BGC-823 and PC-3 tumor bearing mice were injected 0.74–1.11 MBq ^64^Cu-PSMA-617 via the tail vein (*n =* 4). At 1, 4, 24 and 48 h time intervals, the blood was collected by pluck the eyeball before the normal BALB/c mice were sacrificed. Tumor bearing mice were sacrificed at 24 h. The organs of interest were collected, weighted and then the radio-activity was counted with a γ-counter. The data was calculated as the percentage of injected dose per gram of tissue (%ID/g) by comparison with a 1:100 diluted standard dose.

### Micro-PET imaging

PC-3 and BGC-823 tumor bearing mice were injected i.v. with 7.4–14.8 MBq of ^64^Cu-PSMA-617. Blocking studies was performed on BGC-823 tumor-bearing mice by co-injection the radio-tracer with a known inhibitor (S)-2-(3-((S)-1-carboxy-3-methylbutyl)ureido) pentanedioic acid (ZJ-43) (25 mg/kg). Micro-PET imaging was performed at the indicated time points (0.5 h, 1 h, 8 h, 24 h, and 36 h).

For imaging studies, mice were anesthetized with 2.5% and maintained under 1.5% isoflurane (v/v). Imaging was acquired on Super Argus PET/CT (Sedecal, Spain) with the following parameters: 600–900 s PET acquisition time, 80 mm diameter Transaxial FOV, OSEM 3D reconstruction algorithms with attenuation and random corrections. Images were displayed by MMWKS Super Argus.

### Auto-radiography

The tumor tissues embedded in TissueTek (USA) were cut into 25.0 μm thick on a cryostat (Cryo-Star HM 560 MV, Microm). The tumor slides were exposed to a phosphorus plate (Perkin-Elmer, USA) for 24 h. Then the *in vivo* auto-radiographic images were obtained using a phosphor imaging system (Cyclone, Packard).

### Immunohistochemical analysis

Tumor samples were obtained from the PC-3 bearing mice after micro-PET imaging (*n =* 5). Each specimen was formalin-fixed and paraffin-embedded for immuno-assaying. Tissue slices (3.0-mm thick) were dried in 74°C for 30 min and incubated with 3% H_2_O_2_ at room temperature for 10 min. Then sections were put into 0.01 M citric acid buffer for antigen retrieval and microwaved for 20 min. The tissue slices were stained with a rabbit anti-PSMA antibody (ab133579, dilution1:100, Abcam) for PSMA and rabbit anti-CD34 antibody (ab81289, dilution1:100) for CD34. They were incubated in 37°C incubator for 30 min, and then biotinylated anti-rabbit IgG was added onto tumor sections for another 30 min. The sections were also stained with DAB (diaminobezidin). Stained tissue sections were examined under a microscope.

## SUPPLEMENTARY MATERIALS FIGURES AND TABLE



## Abbreviations

PSMA: prostate-specific membrane antigen
ZJ-43: N- [ [ [(1S)-1-Carboxy-3-methylbutyl]amino]-carbonyl]-L-glutamic acid
PET: positron emission tomography
HPLC: High Performance Liquid Chromatography
HSA: human serum albumin
