# Supplementary material for: 64Cu-PSMA-617: A novel PSMA-targeted radio-tracer for PET imaging in gastric adenocarcinoma xenografted mice model
Source: Oncotarget. 2017 May 26;8(43):74159–69. doi: 10.18632/oncotarget.18276 (PMC5650330; doi:10.18632/oncotarget.18276)
Supplement: Supplementary file 1 [file oncotarget-08-74159-s001.pdf]

^{64}Cu-PSMA-617: A novel PSMA-targeted radio-tracer for PET imaging in gastric adenocarcinoma xenografted mice model

SUPPLEMENTARY MATERIALS

In vitro stability study in saline

The *in vitro* stability of ^{64}Cu-PSMA-617 was evaluated in saline (pH 7.4) at 0 min and 28 h post preparation (Supplementary Figure 1). At least three sets of experiments were performed for each time point. HPLC analysis was applied. The column used for HPLC analysis was YMC-Pack ODS reversed-phase column (5 µm, 250 mm × 4.6 mm). The HPLC condition used was 15% to 60% acetonitrile in water with 0.1%TFA within 10 min. *In vitro* stability of ^{64}Cu-PSMA-617 in saline was also evaluated (pH= 7.4) at 28 h post preparation analyzed by ITLC (n = 3). Both methods demonstrated the stability of ^{64}Cu-PSMA-617 in saline.

Electrophoresis analysis of radio-tracer

Using Whatman3 filter paper (length = 20 cm) as carrier, 0.05 mol/L phosphate buffer (pH = 7.4) as solution, ^{64}Cu-PSMA-617 was point in the middle of the paper. Then electrophoresis at 200 V for 4 h to see the final position of the radioactivity.

In vitro cell binding assay

Confluent cells were detached with 0.25% trypsin-0.53 mM EDTA solution, washed twice and re-suspended at the concentration of 2 × 10^6 cells per mL. 24–48 h prior to the binding assay, aliquots of the cell suspension (0.5 mL) were added to each well in the 24-well plate. 37 kBq/mL (1 µCi/mL) of ^{64}Cu-PSMA-617 was added to each well. To determine the specific uptake, the selected wells were treated with the inhibitor ZJ-43 for blocking. After incubation at 37°C for 5, 30, 60 and 120 min, cells were washed twice with 1 mL ice-cold PBS to terminate the cellular uptake, trypsinized with 0.3 nM NaOH buffer. Then, the NaOH solutions were collected and counted in a γ-counter. All radioactivity values were converted into percentage of incubated dose per million cells (%ID/10^6cells). Experiments were performed in triplicate (Supplementary Figure 3).

Quantification of micro-PET images

After i.v. injection of 7.4-14.8 MBq ^{64}Cu-PSMA-617, PC-3 and BGC-823 tumor-bearing mice were anesthetized, then Micro-PET Imaging was performed. Blocking studies were also performed by co-injection with a known PSMA inhibitor (S)-2-(3-((S)-1-carboxy-3-methylbutyl)ureido) pentanedioic acid (ZJ-43) (25 mg/kg) on BGC-823 and PC-3 tumor-bearing mice. Quantification of PET images based on region of interest (ROI) was analyzed. Supplementary Figure 4 shows the mean per pixel values of each PET images over salivary glands and tumor tissues.

Biodistribution

Normal BALB/c mice, BGC-823 and PC-3 tumor bearing mice (n = 4) were injected 0.74–1.11 MBq ^{64}Cu-PSMA-617 *via* the tail vein. After 1 h, 4 h, 24 h and 48 h the blood was immediately collected from normal BALB/c mice, and 24h from BGC-823 and PC-3 tumor bearing mice. Then, the animals were sacrificed. The heart, liver, spleen, kidney, muscle, brain and tumor were collected, weighted and the radioactivity counted as %ID/g by comparison with a 1:100 diluted standard dose. All animal experiments were carried out following the guidelines of the institutional animal ethics committee. The results were shown in Supplementary Figure 5 and Supplementary Table 1.

Mass spectrometry analysis of the decayed product of ^{64}Cu-PSMA-617

The radio-tracer ^{64}Cu-PSMA-617 was characterized by matrix-assisted laser desorption ionization time of flight mass spectrometry (MALDI-TOF-MS) after ten half lives of decay at 4°C. The samples were diluted to 1.0 µg/ml by 0.1 % trifluoroacetic acid (TFA) in water. Sinapinic acid was dissolved in acetonitrile/water/TFA(50/50/0.1) solution to a concentration of 10 mg/ml, and this solution was used for MALDI. 1 µL solution, containing 1:1 mixture sample and matrix, was used for mass analysis. The spectrum was acquired in a positive linear mode and analyzed using the FlexAnalysis v3.0 software (Supplementary Figure 6).

Supplementary Table 1: Biodistribution data of the interested organs of BGC-823,PC-3 tumor bearing mice and normal mice after 24 h injection of ^{64}Cu-PSMA-617 (n = 4)

	BGC-823 tumor xenografted nude mice	PC-3 tumor xenografted nude mice	Normal BALB/c mice
blood	2.7223 ± 0.8941	3.7720 ± 1.1626	1.9106 ± 0.0872
Heart	3.3060 ± 0.3907	3.3002 ± 0.6353	1.8563 ± 0.8962
liver	10.2604 ± 1.5137	9.3429 ± 1.5847	8.1288 ± 1.0843
spleen	3.144 ± 0.22715	2.9295 ± 1.1300	1.9638 ± 0.7046
kidney	5.9121 ± 0.3572	4.2586 ± 0.0835	3.0131 ± 1.2677
muscle	0.9121 ± 0.3572	0.9560 ± 0.1174	1.0874 ± 0.5055
brain	0.5550 ± 0.2302	0.3700 ± 0.0594	0.5994 ± 0.2088
tumor	1.8070 ± 0.2867	3.4658 ± 0.4844	—
T/B	3.4455 ± 0.9130	9.3807 ± 0.1955	—
T/M	2.2123 ± 1.1806	3.6845 ± 0.9591	—

Data are expressed as percentage injected dose per gram of tissue (%ID/g).

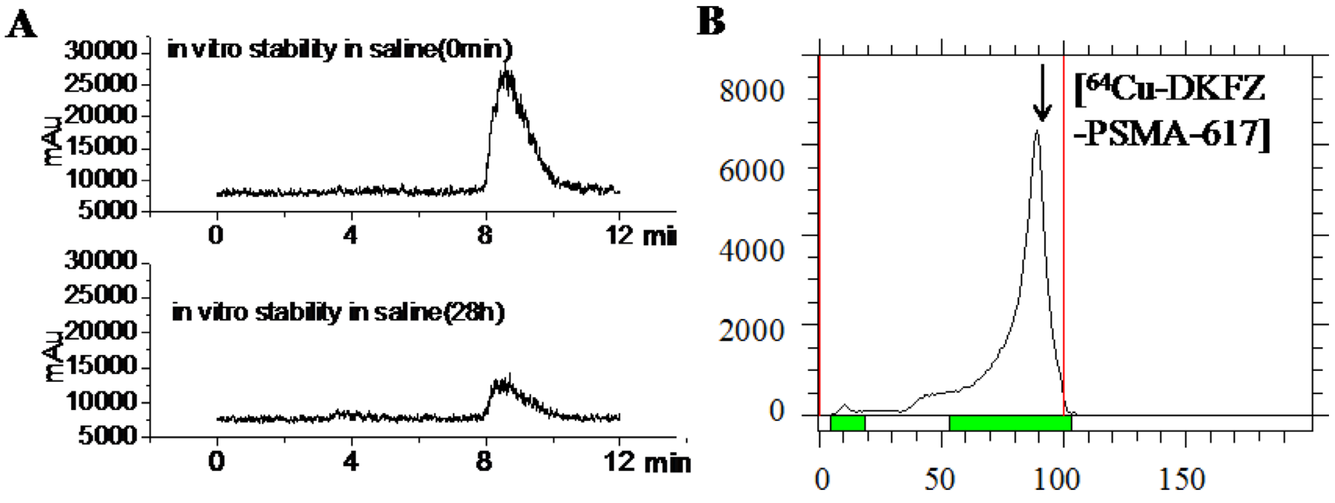

Supplementary Figure 1: Radio-HPLC(A) and ITLC(B) analysis of *in vitro* stability in saline of ^{64}Cu-PSMA-617. (**A**) Stability test at 28 h compared with pure standard, analyzed by HPLC (n = 3). (**B**) Stability test at 28 h analyzed by ITLC (n = 3).

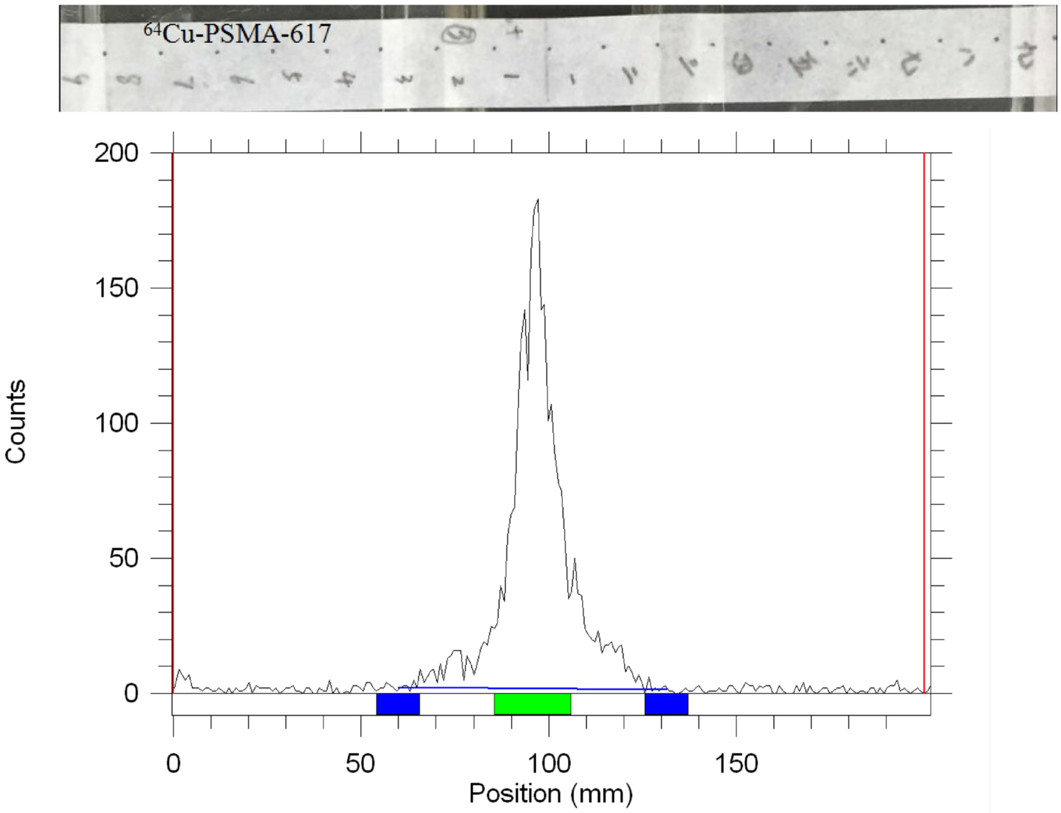

Supplementary Figure 2: Electrophoresis experiment of ^{64}Cu-PSMA-617.

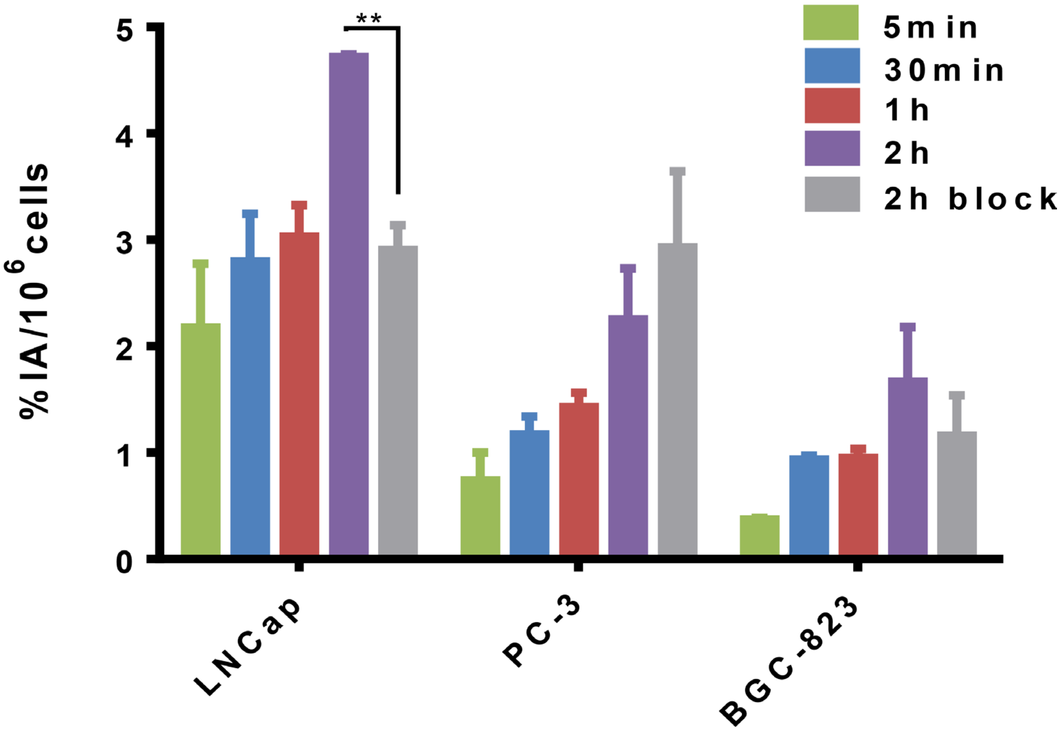

Supplementary Figure 3: Cell binding results of ^{64}Cu-PSMA-617 of PSMA(+) LNCaP, PSMA(-) PC-3 and BGC-823 cell lines in 5 min, 30 min, 1 h and 2 h. (**$P < 0.01$) ($n = 4$).

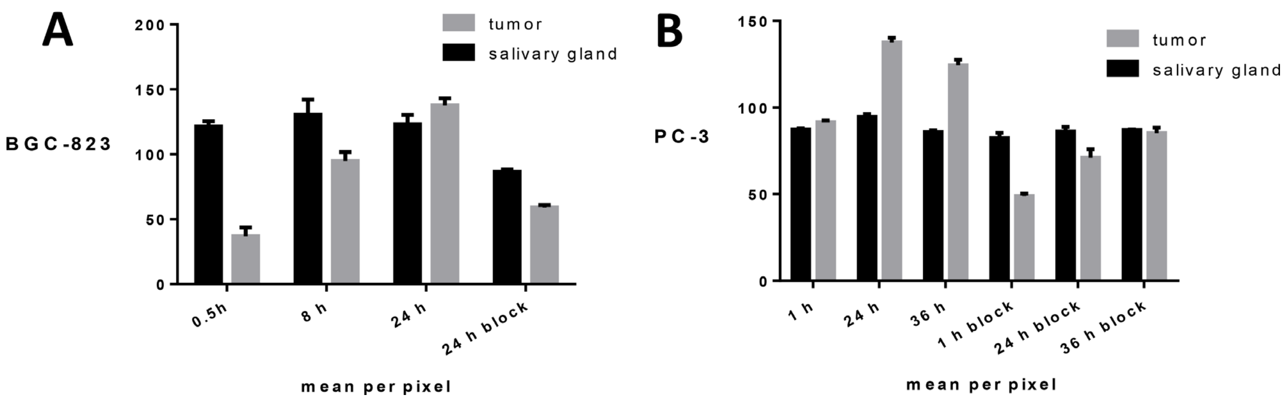

Supplementary Figure 4: Mean per pixel values of BGC-823 and PC-3 tumor bearing mice.

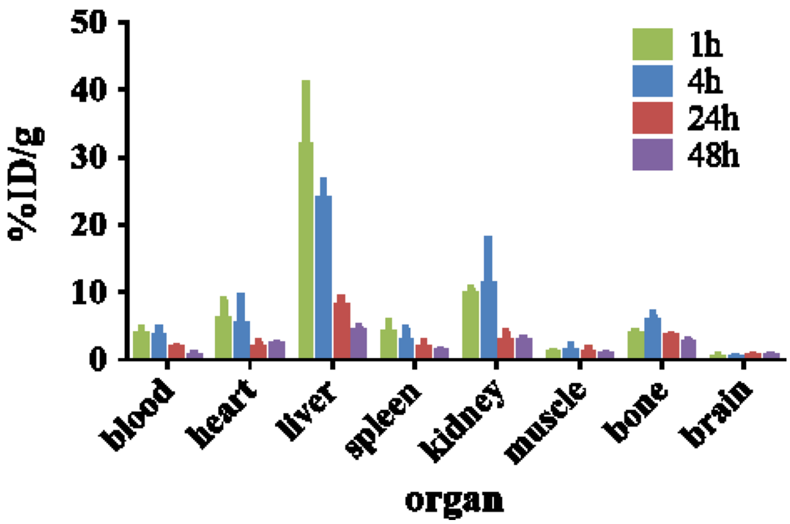

Supplementary Figure 5: Biodistribution of various organs of normal BALB/c mice in 1 h, 4 h, 24 h and 48 h after injection of ^{64}Cu-PSMA-617. (n = 4) The error bar was calculated as the standard deviation.

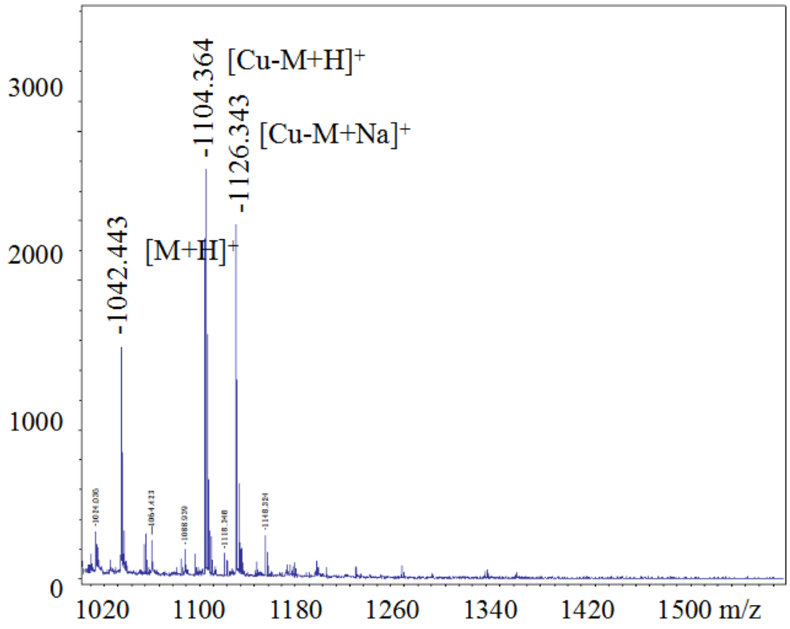

Supplementary Figure 6: MALDI-TOF mass spectra of the decayed product of ^{64}Cu-PSMA-617.
